# All-Optical Nonlinear Real and Fourier-Space Shaping with All-Dielectric Fano Resonant Metasurfaces

**DOI:** 10.1021/acsnano.5c16823

**Published:** 2026-02-06

**Authors:** Falco Bijloo, Masha Ogienko, Arie J. den Boef, Peter M. Kraus, A. Femius Koenderink

**Affiliations:** † 55952 Advanced Research Center for Nanolithography, Science Park 106, 1098 XG Amsterdam, The Netherlands; ‡ Department of Physics of Information in Matter and Center for Nanophotonics, NWO-I Institute AMOLF, Science Park 104, 1098 XG Amsterdam, The Netherlands; § Department of Physics and Astronomy, and LaserLaB, Vrije Universiteit, 10810 HV Amsterdam, The Netherlands; ∥ 530573 ASML Netherlands B.V., 5504 DR Veldhoven, The Netherlands

**Keywords:** nonlinear, metasurface, all-optical, beam, shaping, Fano, Fourier

## Abstract

A main goal within the metasurface community is to develop dynamic, ultrafast tuning strategies for controlling beam profiles and directionality, especially in the ultraviolet regime. We present all-optical nonlinear beam shaping in both the beam profile and its angular distribution. We use a digital mirror device within a pump–probe setup that allows spatial pump patterns of a visible light pulse to spatiotemporally coincide with an infrared probe pulse onto an all-dielectric Fano resonant metasurface. The infrared pulse is tuned near the Fano resonance to generate strong third harmonics, and the pump pulse locally deactivates harmonic generation due to excitation of carriers that broaden and blue-shift the resonance. In Fourier space the spatially periodic pump patterns convolves with the third-harmonic diffraction pattern, which generates satellite orders that evidence coherent emission and directional control. This work enables ultrafast, precise control over harmonic beam profiles and directionality at the generation stage.

## Graphical abstract

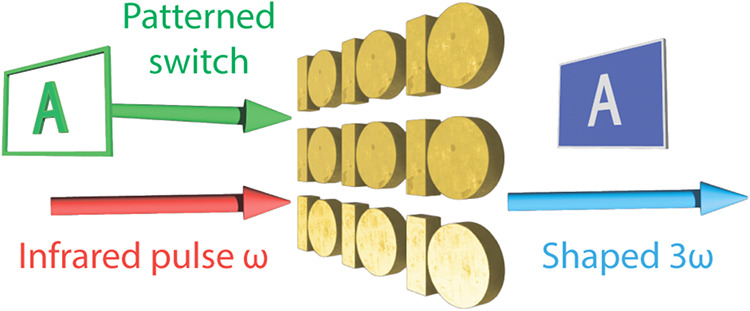

Nonlinear all-dielectric metasurfaces have enjoyed increased attention over the past decade.^1,2^ By harnessing the strong field enhancement inside meta-atom particles, metasurfaces can achieve harmonic generation with conversion efficiencies orders of magnitude higher than those of unstructured films, resulting in exceptionally bright emission,^3^ while eliminating phase-matching constraints^4,5^ and allowing tailorable wavefronts that are imprinted by nanostructure design.^6^ These advantages have been demonstrated across a range of nonlinear processes, including second-harmonic generation (SHG),^7,8^ third-harmonic generation (THG),^9,10^ four-wave mixing (FWM),^11^ and high-harmonic generation (HHG).^12,13^ The required strong field enhancements are generally achieved by designing metasurfaces to support (interfering) Mie modes.^14,15^ High-quality factor (*Q*) Fano resonances can arise from the interference between bright and dark modes.^16^ These Fano resonances are shown to enhance nonlinear conversion efficiencies significantly.^17^ Common approaches include coupling a dark mode to a bright mode via the near-field,^3^ or breaking symmetries to introduce radiative leakage into otherwise nonradiative, forbidden modes, a mechanism also referred to a quasi-bound states in the continuum (q-BIC).^9,13,18,19^

A key feature of nonlinear metasurfaces is diversity in functionality. Nonlinear effects, such as harmonic generation, add a plethora of possibilities to shape and control light emission.^2^ This is especially relevant for imaging and microscopy using sources at ultraviolet (UV) wavelengths, where beam shaping by conventional linear optical components is difficult and often fails to perform effectively.^20,21^ Shaping the Fourier space, namely, controlling the directionality of light emission, has become a critical area of research,^10,22^ next to the generation of orbital angular momentum beams,^23^ vector vortex beams^24^ and structured light.^25,26^ Even though metasurfaces offer these diverse solutions, one main drawback is that the metasurface function is fixed at fabrication. Over the past decade, tunable metasurfaces have been explored as a solution to counter the fixed functionality,^27^ for instance, based on thermo-optical effects,^28^ electric biasing,^29^ liquid crystals,^30^ interferometric routing,^31^ and mechanical tuning.^32^ Electro-optical modulators offer the ability to achieve the desired very high operation speeds,^33,34^ but often compromise on modulation contrast^35,36^ and do not allow for local switching. In general, there is a trade-off for many tunability mechanisms to either tend to be slow, work at low modulation contrast, or offer little room for desired wavelength ranges,^37^ such as for shaping UV beams. Consequently, there is a significant ongoing effort within the metasurface community to develop dynamic tuning strategies capable of ultrafast control over beam profiles and directionality with large contrast, particularly in challenging and desired wavelength regimes.

In this article, we present all-optical, ultrafast, high-contrast nonlinear beam shaping in both real space (nonlinear source beam profile at the metasurface) and Fourier space (directionality). We use a digital mirror device (DMD) within a pump–probe setup that allows spatial patterns to be imprinted on a pumping (or switching) pulse, which spatiotemporally coincides with an infrared (IR) probe pulse onto an all-dielectric Fano resonant metasurface. The IR pulse is tuned near the Fano resonance to generate strong third harmonics (TH). The pump light (pulses chosen in the visible area in this work) projects an image of the DMD onto the metasurface. This pump pulse locally deactivates harmonic generation due to excitation of carriers that broaden and blue-shift the resonance.^38^ Nonperiodic pump patterns (such as alphabetic characters) are successfully imprinted onto the TH beam, demonstrating spatial wavefront control. Furthermore, we show using spatially periodic and aperiodic pump patterns that the angular distribution of TH emission convolves with the structured pump’s spatial frequency components. The convolution produces satellite diffraction orders that evidence coherent emission and enable directional control. We found that the spatial programming of the pump is effective down to a minimum resolution of a single meta-atom, which is necessary to trigger harmonic deactivation. However, the deactivation mechanism features a transition region spanning about two meta-atoms. Only beyond this spatial threshold is full deactivation reached, which we attribute to the intrinsically nonlocal nature of the Fano resonance. It is a striking and counterintuitive result that an intrinsically nonlocal metasurface resonance can be locally switched with such large contrast and with such a fine resolution of just ca. 2 unit cells. Furthermore, by controlling the time delay between the IR probe and visible pump pulse, we reveal the linear transient resonant response of the metasurface and its impact on the THG conversion efficiency. These transient observations are closely replicated by a temporal coupled-mode theory (TCMT) model. Our findings demonstrate a powerful platform for all-optical dynamic beam shaping with nonlinear metasurfaces. These results enable applications in free-space optical communication, dynamic holography, and ultrafast reconfigurable photonic components, such as adaptive lenses, tunable filters, and beam splitters, or can be used in integrated photonic circuits for high-speed modulation and routing. Furthermore, as this method holds for any harmonic order, it opens the door to ultrafast, precise control over UV beam profiles and directionality at the generation stage.

## Results and Discussion

The main concept of our experiment is visualized in Figure 1. A 130 fs IR (1480 nm) pulse excites a Fano resonant metasurface, thereby generating a third harmonic emission. The metasurface consists of silicon meta-atoms comprising a disk adjacent to a bar on quartz. The disk-bar meta-atom structure has been studied extensively, also in the context of third and high-harmonic generation.^3,12^ The Fano resonance appears due to the interference of a bright electric dipole (ED) mode that is associated with the long axis of the bar and a dark magnetic dipole (MD) mode pointing out of the plane that arises from circulating displacement currents in the disk. The strong field enhancement of the resonance allows for a large harmonic generation efficiency.^39^ By means of a separate beam path that passes through a delay stage and reflects from a digital mirror device (DMD), a 515 nm pump pulse projects a spatially programmable pump pattern onto the metasurface. As the pulse carries light with an energy that is larger than the band gap of silicon, part of the pump light is absorbed by the nanoparticles, which excites carriers to the conduction band. The excited free carriers are associated with a refractive index change, which in turn blue-shifts and broadens the resonance,^38,40^ reducing the THG efficiency. Since in this scenario the pump light absorption is associated with a reduction in THG generation, the negative of the pump pattern is transferred into the TH emission.

**Figure 1:**
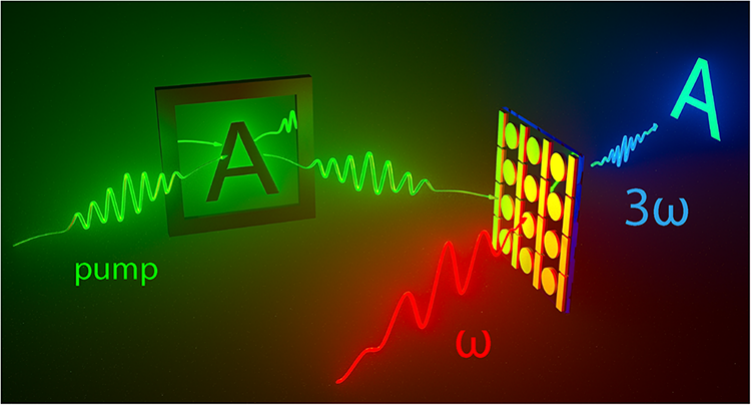
Illustration of our scheme to optically shape nonlinear beam profiles and control directionality. An IR pulse (ω, red) illuminates the sample to generate bright TH (3ω, blue) from a Fano resonant disk-bar metasurface. A second pulse (pump, green) reflects from a DMD to project a spatial pump pattern on the metasurface that is negatively imprinted onto the TH emission profile. Credit: the illustration was designed by FB and Max Postma (AMOLF design department) and realized by Max Postma. It is used with permission.

We fabricated disk-bar metasurfaces in polycrystalline silicon (135 nm thickness) that was evaporated onto fused quartz via e-beam evaporation, and patterned with e-beam lithography. The meta-atoms are arranged in a square grid of 900 nm pitch, with each unit cell containing one disk of 240 nm radius adjacent to an 800 mm × 205 nm bar (scanning electron micrograph in the inset of Figure 2a). The Supporting Information (SI) of ref 38 reports in detail on the nanofabrication procedure. Our experimental setup allows for exciting the metasurface with minimum angular spread, by using a long focal distance lens (*f* = 200 mm, Thorlabs) that focuses in the back focal plane (BFP) of a microscope objective (Nikon 50×, NA 0.8), effectively demagnifying our parallel IR beam to a radius of 20 μm. By using the rather broad bandwidth of the 130 fs pulse, linear infrared transmission measurements (presented in Figure 2a, solid black) reveal a narrow Fano resonance with a quality factor *Q* ≃ 500, with light polarized along the long axis of the bar, measured by a grating-based spectrometer equipped with an InGaAs detector (Avantes, AvaSpec-NIR256/512–1.7-HSC-EVO). The bright ED mode is visible in the transmission spectrum (Figure 2a) as the broad dip at 1550 nm and the dark MD mode as the narrow dip at 1490 nm.

**Figure 2:**
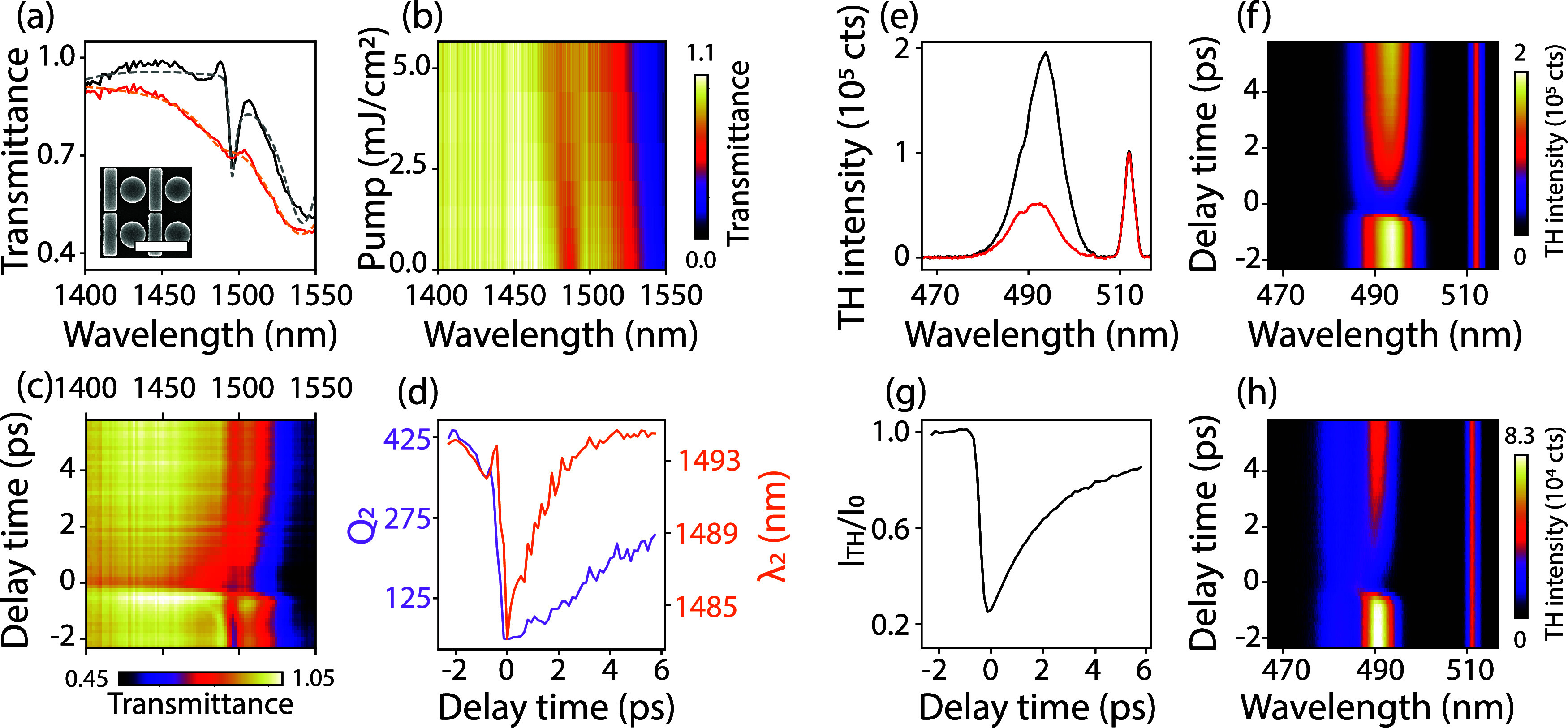
Transient Fano modulation and TH deactivation. (a) Linear transmittance through a disk-bar metasurface (SEM inset, scale bar 1 μm). A Fano resonance of *Q* ≃ 500 appears at 1490 nm that is apparent without pumping (solid black) and disappears with pump (solid red), at time *t*_0_ and pump fluence = 2.52 mJ/cm^2^. The dashed curves are coupled oscillator fits to the data in order to extract *Q* and the resonant wavelength. (b) Transmittance as a function of pump fluence on the vertical axis. (c) Transient transmittance at pump fluence 2.52 mJ/cm^2^, with pulse delay time on the vertical axis. (d) Quality factor of the Fano *Q*_2_ (purple) and the resonant wavelength λ_2_ (orange). (e) Emitted TH spectrum at *t* < *t*_0_ (black) and at *t* = *t*_0_ (red), showing a clear reduction of THG efficiency. Residual pump light that is not filtered out by the notch filter is visible as the peak near 515 nm. (f) Transient TH spectrum for a probe wavelength of λ = 1480 nm. (g) TH deactivation obtained by spectrally integrating the TH spectrum from 490 to 500 nm. (h) Transient TH spectrum for a nonresonant probe pulse of wavelength λ = 1440 nm, showing THG enhancement at the Fano resonance centered at 1490 nm. Pumping visibly induces a blue shift at *t* = 0 ps.

We use a pump–probe configuration with a delay stage to precisely control the time delay between a 515 nm pump and an IR probe. The experimental setup is described in detail in the Methods. The 515 nm pump pulse reflects off a DMD to imprint spatial pump patterns. Figure 2a shows a transmittance spectrum (red solid line) with pump and probe pulses overlapping in time and space, at a pump fluence of 2.52 mJ/cm^2^. Here, the DMD is set to operate as a mirror, and we have precisely tuned the pump power and delay time for temporal overlap. In this switched configuration, no trace of the Fano resonance is evident. In ref 38, we hypothesized and validated via simulations, based on earlier work on ultrafast switching of silicon photonic structures, that the pump light is directly absorbed in the silicon. This absorption leads to free carriers that modulate the refractive index, which, in ref 38, we described with a Drude model. In this picture, one expects a reduction of the real part of the refractive index *n*, and an increase in the imaginary part *k*. According to electromagnetic simulations, this refractive index change in turn causes a blue-shift of the metasurface resonances, accompanied by a deterioration of the quality factors due to free carrier absorption at infrared wavelengths. Recent work shows similar blue-shifting optical resonance control via light-induced pumping.^41^ The experiment presented in Figure 2b examines the dependence of this resonance modulation as a function of pump fluence with the delay time fixed at *t*_0_, measured by an optical spectrum analyzer (Thorlabs, OSA202C). Temporal overlap *t*_0_ in this work was determined by maximum harmonic deactivation (see discussion below of the THG signal). The observed broadening and shift in the linear IR transmission of both the narrow and broad resonant modes confirm our hypothesis, consistent with the direct absorption of the pump pulse and subsequent carrier excitation.

Having confirmed the pump-induced resonance shift and broadening at temporal overlap, we next investigated the transient dynamics of the metasurface response. Transient changes in the resonant behavior of the metasurface are studied by keeping the pump fluence fixed while scanning over the delay time. Figure 2c presents a time trace of 8 ps in steps of 133.3 fs, at pump fluence 2.52 mJ/cm^2^, captured with the same spectrometer as Figure 2a. At the onset of *t*_0_, a very strong broadening and shift are observed in the transmittance spectrum. This effect is maintained for at least a few picoseconds, likely determined by thermalization and carrier recombination times in the nanostructured polycrystalline silicon, next to carrier diffusion from hotspots to the rest of the nanoparticles.^42–44^ The long-lasting dynamics evidence the absorptive nature of the effect rather than nonlinear effects that solely occur instantaneously. The transient blue-shifting behavior of the resonance under pumping is, again, similarly observed by recent work.^41^ Ultrafast dynamics are visible before the onset of *t*_0_. While the pump pulse arrives after the probe pulse, it arrives within the long ring-down of the polarization inserted by the probe into the long-lived resonance (∼0.4 ps). The pump pulse can couple this tail of the induced polarization to other frequencies. Comparable pre-*t*_0_ dynamics have been reported in refs 45–47 . We investigate the transient response of the fundamental resonance via a temporal coupled-mode theory model,^45,48,49^ which is reported in detail in the Supporting Information (SI). The resonance shift, broadening, and pre-*t*_0_ dynamics that are observed in the experimental transient response are closely replicated by the model (Figure S4.2). These effects have interestingly been studied in detail by other domains, such as EUV spectroscopy of (autoionizing) transitions in noble gases,^50^ molecules,^51^ and even solids.^52^ Furthermore, transient IR probe spectra are found in the SI, as well as the normalized IR transmittance ratio *T*/*T*_0_, where *T*_0_ is the transmittance spectrum at *t* = −2 ps to highlight pump-induced changes. The shift and broadening of the resonance are investigated by fitting a coupled oscillator function (example fits in Figure 2a, dashed lines) to the transmittance spectrum at each time step. The time trace of the fitted quality factor *Q*_2_ and resonant wavelength λ_2_ of the dark mode is presented in Figure 2d. The quality factor is indeed greatly reduced (ca. 3-fold reduction), while the resonant wavelength blue-shifts at a time *t*_0_ of about 7 nm and recovers over picoseconds. The samples are stable under long-term pumping. No change of signal was observed for pumping at 5 mJ/cm^2^ for half an hour. However, pump fluences greater than 10 mJ/cm^2^ resulted in irreversible damage to the sample.

Panels e–h in Figure 2 present transient TH deactivation measurements. Details of the nonlinear deactivation experimental setup are presented in the Methods. The IR probe pulse is centered near the Fano resonance to increase the THG conversion efficiency and operates at a fluence of 0.2 mJ/cm^2^, maximizing THG conversion without reaching saturation. Typical THG conversion efficiencies are η ≃ 10^–6^ to 10^–5^. Figure 2e presents two TH spectra, where the black spectrum shows the regular TH spectrum without pump and the red spectrum shows the TH spectrum under pumping at *t*_0_ (pump fluence 2.52 mJ/cm^2^). The THG efficiency is greatly reduced due to the resonance being pumped out of spectral overlap with the IR probe pulse and the introduced loss of resonance by excited free carrier absorption. We scan the same time delay range as in Figure 2c, to study the transient TH deactivation. Figure 2f shows almost 80% deactivation of THG at *t*_0_, slowly recovering the signal over picoseconds. Similar behavior is replicated by the TCMT model that is presented in the SI (Figure S4.2). The model yields a narrower THG spectrum, as it accounts only for the contribution from the narrow Fano resonance. In reality, the situation is more complex, as the broader bright resonator also contributes to the THG, resulting in a broader TH spectrum. Moreover, to fully describe the transient dynamics, one must account for interference between these two modes, which further enriches the observed behavior.^53^ By integrating over a narrow spectral band (490–500 nm), we study the total dynamic TH suppression and the associated time scales (Figure 2g). This relatively large modulation is consistent with previous work,^38^ which reported even higher modulation strengths. In general, the modulation strength can be optimized by adjusting parameters such as pump fluence, pulse tuning and length, and polarization. However, such optimization is not the topic of the present work, and we consider a modulation depth of approximately 80% to be sufficient for our purposes. To further evidence the dominant contribution of the Fano resonance to the THG efficiency, we chose an IR pulse that is spectrally blue-shifted (centered at ∼1450 nm) from the Fano resonance (∼1490 nm). Figure 2h shows a TH spectrum time trace where two key observations can be made. First, even though the central wavelength of the IR pulse is at 1450 nm (= 485 nm × 3 nm), the strongest THG occurs in the spectral tail of the pulse near the Fano resonance at 496 nm (496 nm × 3 nm = 1490 nm).^9,54^ Second, at time zero, the peak of the THG is blue-shifted and is even enhanced for a small bandwidth, before slowly returning to its original wavelength. Similar blueshift and enhancement behavior is reproduced by the TCMT model for a blue-detuned driving pulse (Figure S4.3) in the SI. These two experimental observations, together with the reproduction by the model, further evidence the spectral overlap dependence and the effect of the blueshift of the resonance on the THG efficiency.

The previous measurements reveal the linear and nonlinear dynamic metasurface responses to uniform excitation. Next, we explore how the spatial structuring of the pump field affects the nonlinear emission. We use the DMD to project binary spatial patterns, i.e., fully black and white intensity patterns without intermediate gray levels. These binary patterns impose a strong spatial modulation on the pump intensity, which, through absorption in the metasurface material, is expected to induce local variations in the meta-atom resonance, enabling control over the spatial profile of the nonlinear emission. Figure 3 shows six examples of dynamically shaped harmonic beams measured using real-space microscopy of the sample at the TH wavelength. For reference, Figure 3a represents the spatial distribution of THG for a fully reflective DMD (no pattern projected, camera integration time: 200 ms). Even though the TH real-space images are slightly out of focus on the camera, the individual unit cells are visible. This results from the fact that although the metasurface is subdiffractive at the fundamental wavelength (1480 nm), the unit cell pitch (900 nm) is well within the diffraction limit for the THG wavelength (490 nm light, NA 0.8, in air). Figure 3b–d displays the pump field on the left in green and the corresponding third harmonic real-space image in blue on the right, at pump fluence 0.63 mJ/cm^2^. In each case, the pump field is shaped as letters A, B, or C, resulting in a TH signal that appears as a bright spot with the respective letter subtracted. (Figure 3e–g) present the results for pumping the inverse of the letters A, B, and C, which are displayed on the left side, with the corresponding TH images on the right, at pump fluence 0.32 mJ/cm^2^. In all TH real-space images, a gradient of TH deactivation is visible from the bottom left to the top right, which is due to a spatially varying *t*_0_. This *t*_0_-gradient results from a small optical path difference across the DMD, caused by the intrinsic mirror tilt (≃10°) along the diagonal of mirrors in the ON state. Because the DMD must be tilted to compensate for this, reflections from one side of the DMD arrive slightly earlier than those from the other end. As estimated, across a millimeter beam width on the DMD (which is 50× demagnified to ca. 20 μm on the sample), a tilt angle of 10 degrees provides a few 100 μm (equivalent to 0.3 ps) optical path difference. The diagonal orientation of the gradient is commensurate with the diagonal orientation of the DMD mirror tilt, and the time difference corresponds to the measured transient suppression that is presented in (Figure 2g). There is a notable width difference in the letters in the deactivated (Figure 3b–d) and activated (Figure 3e–g) cases. This raises the question of the spatial extent of (de)­activation, which will be examined in spatial resolution experiments that are discussed below.

**Figure 3:**
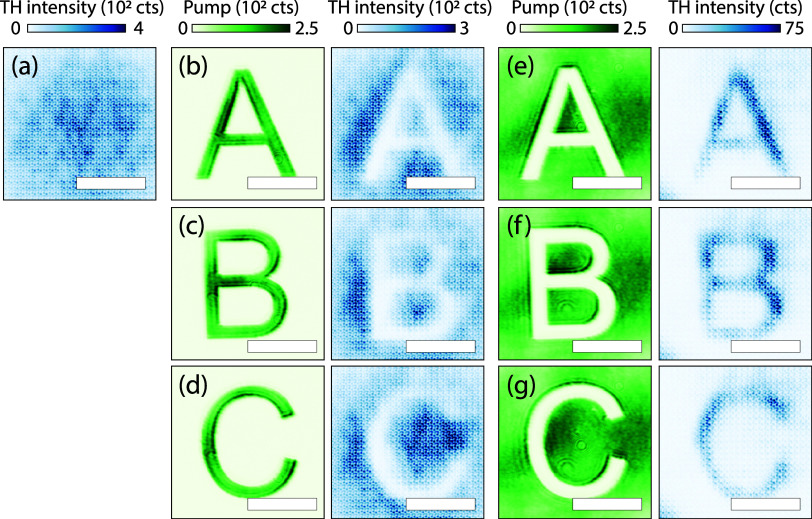
Nonlinear real-space shaping. The obtained TH signal is depicted in blue, while pump patterns are depicted in green, with color bars above the plots. The scale bars depict 10 μm. (a) Measured TH spot without pump pulse. (b) Pump pulse pattern depicting an “A” on the left, generated a TH spot with the letter subtracted on the right. (c) Similar results for B and (d) for C. (e) Pump pulse pattern showing the inverse of (b) on the left, which generates an A in TH signal, with (f) B and (g) C.

A crucial feature of nonlinear metasurfaces is that the harmonic generation is coherent, meaning that spatial structuring in the near field of the sample should also immediately impact the angular distribution of emitted third harmonic light. As such, we envision our method of all-optical spatial structuring to also present a method for all-optical structuring of directionality. This capability is not immediately evident from the real-space images: real-space amplitude modulation by a deactivating pulse can, in principle, be replicated using incoherent sources, such as fluorescence, but in that case, the angular distribution remains unaffected. We demonstrate nonlinear Fourier-space shaping in Figure 4. The pump fluence in this measurement set was maintained at an average of 5 mJ/cm^2^. This chosen fluence is larger than the previous measurement presented in Figure 3, to reduce the effects of the deactivation gradient that is introduced by the spatially varying *t*_0_ via DMD. Fourier-space shaping requires deactivation over long lateral length scales, as spatially separated sources radiate synchronously to provide diffraction. The lateral deactivation strength variation is reduced by saturation at large pump fluences,^38,55^ resulting in a more homogeneous deactivation distribution over a large lateral length scale. A plethora of pump patterns were used to study the angular emission, including hexagonal lattices, two-dimensional (2D) grids, and hyperuniform patterns. Without pumping, we expect a square TH diffraction pattern that consists of discrete nonlinear gratings orders, i.e., discrete points at angles that derive from the TH wavelength λ_TH_ and metasurface pitch *p* through $\frac{k_{x,y}}{k_{0}}=\sin\theta =\frac{m\lambda }{p}≃0.58$ , with *m* the order ±1. A Fourier image confirming this intuition is displayed in Figure 4a. In the pumped cases, we expect a similar diffraction pattern, convolved with the Fourier transform of the (photonegative) of the pump pattern. This follows the logic of X-ray diffraction and antenna array theory translated to the case of nonlinear sources. Following this logic, the near-field nonlinear source distribution is written as the product of the source distribution (a lattice with the metasurface pitch) in the absence of the pump, multiplied by an amplitude mask induced by the switching pattern. Consequently, the far field is expected to be the convolution of the lattice radiation pattern (grating orders) and the transform of the amplitude mask. By way of example, Figure 4b shows a hexagonal hole pump pattern on the left, its generated TH real-space image in the middle, and the Fourier-space angular emission profile on the right in log-scale, to highlight subtle low-intensity differences. Compared with the reference case, hexagonal satellite orders are generated around each diffraction order. In Figure 4c, the hexagonal directionality is generated at a smaller reciprocal lattice vector as the pump pattern (shown on the left) pitch is increased. The 2D square grid, presented in Figure 4d, reveals square satellite diffraction orders. Furthermore, this is not restricted to periodic amplitude masks. For example, in Figure 4e, we use an amplitude mask of patches and veins that is a so-called ‘hyperuniform’ pattern: its Fourier transform presents a ring in Fourier space.^56^ Indeed, THG shows isotropic scattering rings around the grating orders. The set of measurements presented in Figure 4 clearly shows the wide variety of shaping nonlinear directional emission by spatial deactivation with a pump.

**Figure 4:**
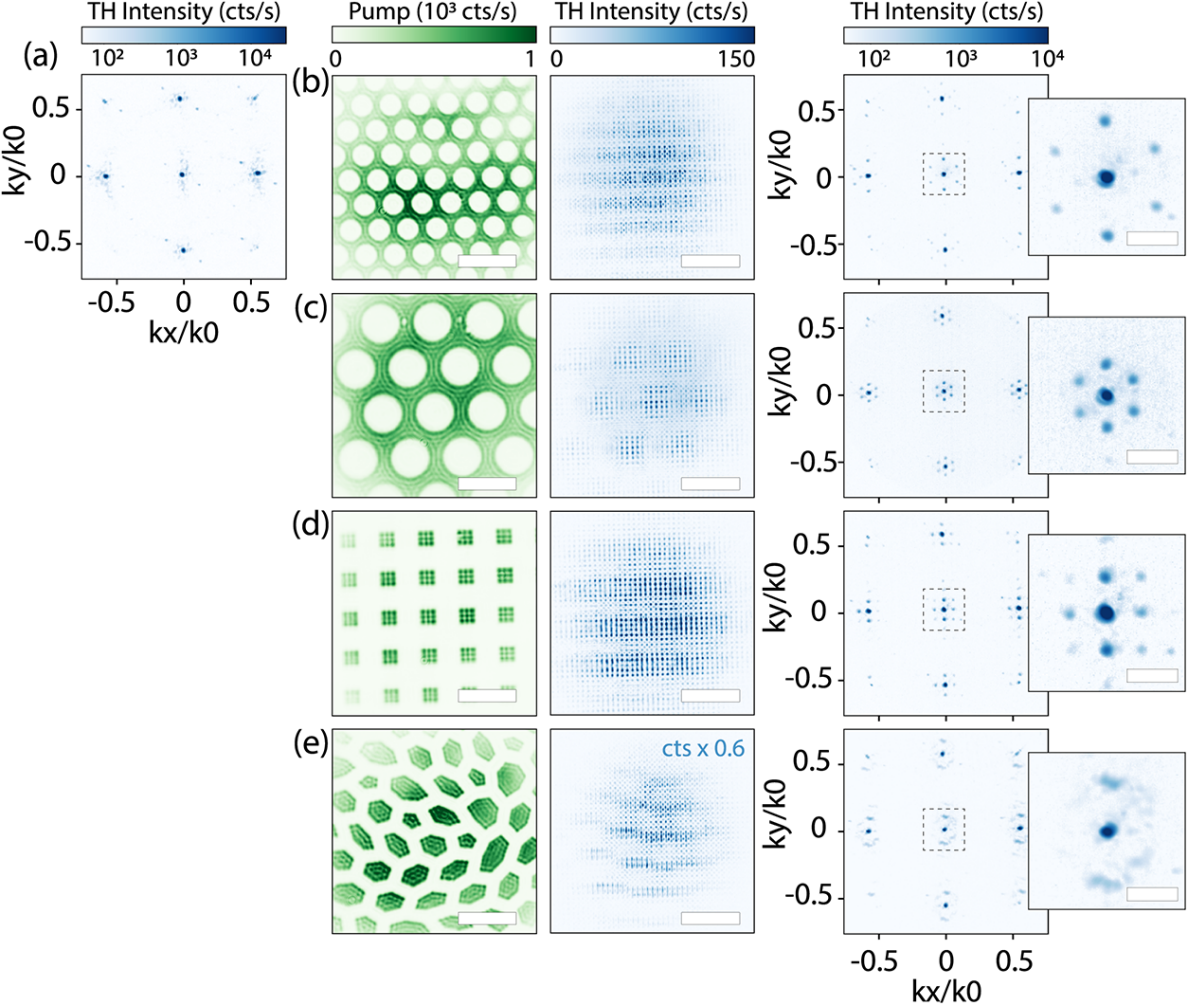
Nonlinear Fourier-space shaping. Scale bar depicts 10 μm. (a) Measured Fourier-space pattern without pump. (b–e) The spatial pump patterns are shown on the left in green, with the accompanying produced TH real (Fourier) space image in the middle (on the right). On the far right is an enlarged Fourier-space image of the dashed area (|*k*_*x*,*y*_/*k*_0_|< 0.155) to highlight changes around *k*_0_, with scale-bar *k*_*x*,*y*_/*k*_0_ = 0.1. The spatial TH deactivation is visible in the real-space image, which produces hexagonal (b, c) or square (d) satellite orders around the metasurface diffracted orders. The hyperuniform pattern (e) produces a scattered ring around all diffraction orders.

Finally, we assess some of the limits of this approach. In particular, we examine the spatial extent or spatial resolution of this form of harmonic deactivation. On the one hand, one might argue that our DMD and pump light combination has the resolution to engage (sub)­single metasurface unit cells. On the other hand, Fano resonances in metasurfaces are nonlocal, suggesting that effectively the spatial resolution is limited to a few unit cells. To address this question experimentally, we produce a set of pump patterns that include a circular window or a circular spot, with variable radius, and a periodic 2D square checkerboard pattern with variable gap line width. The goal of this exercise is to extract the minimum number of meta-atoms necessary for harmonic generation or deactivation. Figure 5a shows the circular window (spot) pump pattern on the left (right) side, at radius *r* = 2.3 μm. In this measurement set, the pump fluence was kept at an average of 4.41 mJ/cm^2^, and the time was at *t*_0_. Examples of produced TH real-space patterns are displayed in Figure 5b, for the window (spot) on the left (right) side at *r* = 3.8 μm. We study the TH brightness by integration of the counts inside the pump area, which is indicated by the black dashed circle in Figure 5b. The square of the integrated counts as a function of pump radius is presented in Figure 5c. At *r* > 0.9 μm (indicated by the vertical dashed line at meta-atom 2), the square of the TH integrated intensity for both the window or spot logically follows a straight line, as the TH counts grow as the square of the area. The slope of the TH light as a function of pump radius is determined by deactivation (spot, purple) or no deactivation (window, orange). At values *r* < 0.45 μm (indicated by the area left of the vertical dashed line called meta-atom 1), the TH intensity within the window area (orange markers) increases only slowly, indicating that the radius is insufficient to activate harmonic generation. In the same range, the integrated TH intensity in the spot area (purple markers) exhibits a steeper increase compared to the region beyond *r* > 0.9 μm. A clear change of slope in integrated TH intensity for both pump patterns is observed between *r* = 0.45 μm and *r* = 0.9 μm. These observations indicate that a minimum of >1 meta-atom (*r* = 0.45 μm, at metasurface pitch *p*_m_ = 900 nm) is necessary for resonance modification by pumping, whereas the nonlocal nature of the resonance is maintained at more than 2 meta-atoms. This result is further supported by analyzing a cross-cut (transversely summed over 3 μm) of the TH real-space image, presented in Figure 5d, which reveals the number of unit cells involved in the transition from the activated to the deactivated region. Especially in the vertical cross-cuts, displayed in the bottom two graphs, a transition is visible of 1–2 unit-cells. A possible limiting factor to the observed spatial extent is the optical resolution of the projected pump pattern itself. The experiments presented in Figure 5a show an edge transition width of about 373 nm, which is consistent with the diffraction limit of our NA = 0.8 objective (Methods), and far below the transition region (>900 nm). This edge width indicates no significant contribution to the observed transition of 1–2 unit cells.

**Figure 5:**
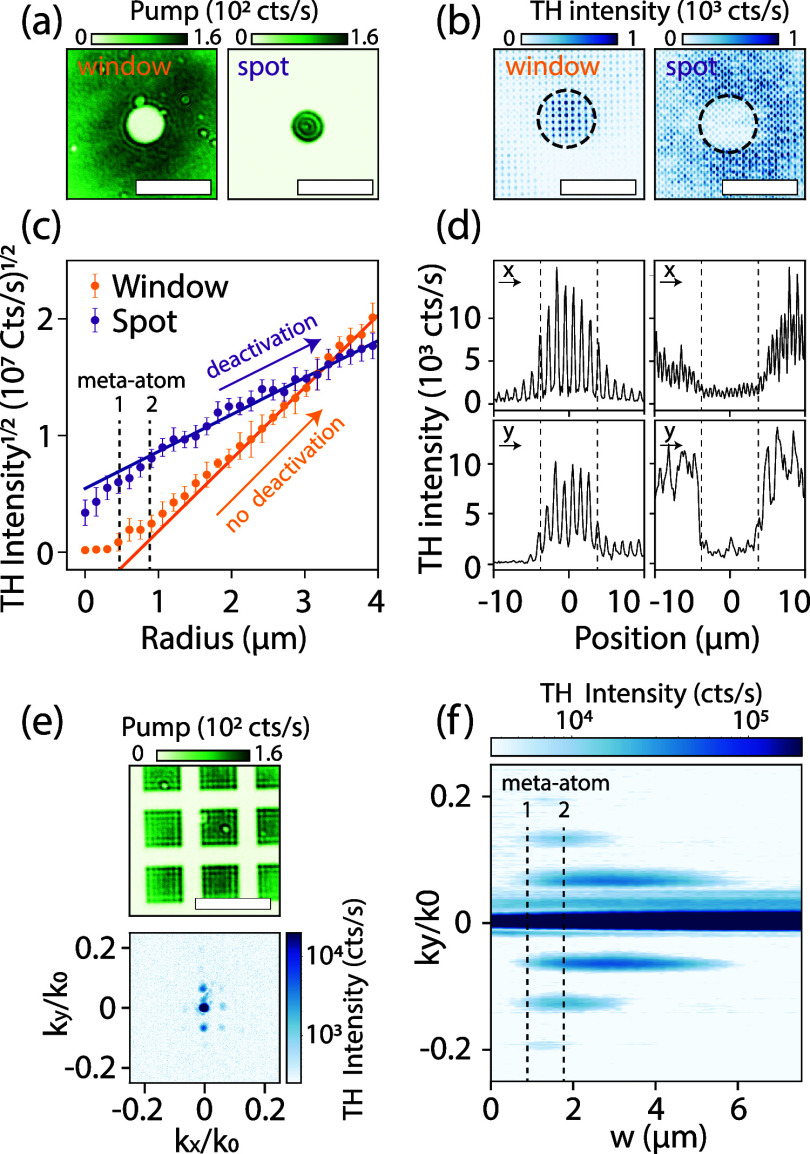
Spatial TH deactivation extent study. (a) Pump image of the window (spot) at radius *r* = 2.3 μm on the left (right), scale bar depicts 10 μm. (b) The TH real-space images for the window (spot) on the left (right) at radius *r* = 3.8 μm, with integration boundary indicated by the dashed black circle. (c) The square root of the TH intensity in the integration area for window (orange markers) and spot (purple markers) as a function of pump radius, with linear fits to the region *r* > 1.5 μm (solid lines). Error bars show the standard deviations over 10 repeated measurements. The vertical dashed lines indicate where the diameter spans 1 and 2 meta-atoms. (d) TH intensity cross-cuts summed over a band of 3 μm in the center of the window (spot) on the left (right), where the horizontal (vertical) direction is on the top (bottom). Dashed vertical lines indicate the pump area region of *r* = 3.8 μm. (e) (top) 2D checkerboard pump pattern at gap line width *w* = 2.3 μm (bottom) generates square satellite orders around the metasurface diffraction orders in TH Fourier space (image taken at gap line width *w* = 3.8 μm). (f) Vertical TH Fourier cross-cut summed over a small band of −0.008 < *k*_*x*_ < 0.008 as a function of pump checkerboard gap line width *w*. Dashed vertical lines indicate where *w* spans 1 and 2 meta-atoms.

In addition to the real-space spatial extent analysis, we also examine the system’s behavior in Fourier space, providing complementary insight into its momentum-resolved characteristics. Figure 5e shows the periodic 2D checkerboard pump (top, example image at *w* = 2.3 μm) and the TH Fourier image (bottom, at *w* = 3.9 μm). We focus on the satellite diffraction orders produced at around *k*_0_. Figure 5f tracks a vertical cross-cut of the TH intensity (summed over a band of −0.008 < *k*_*x*_ < 0.008), as a function of the gap line width between checkerboard blocks *w*. No satellite orders are created below pattern line widths *w* < 0.9 μm (indicated by vertical dashed line meta-atom 1), whereas the TH intensity starts leaking into the satellite diffraction orders at gap line widths of *w* > 0.9 μm, which is consistent with the previous estimate that at least a single meta-atom is required to (de)­activate harmonic generation. To confirm that this observation is really attributable to the modulation mechanism, and not to a property of linear diffraction theory, in the SI, we show calculated diffraction efficiencies according to linear diffraction theory for binary grids (i.e., supposing that the modulation has subsingle meta-atom resolution). In this case, satellite orders already appear at *w* < 0.9 μm. We can thus conclude from our observation that the nonlocal nature of the Fano resonance places a physical limit on the modulation resolution. The diffraction efficiency into the satellite orders as compared to the main order is reported in the SI (Figure S3.4), and is at the level of a few percent for a single diffraction order. This is in excellent accord with Fourier optics theory (structure factor form factor analysis) for the same patterns, for which calculations are reported in SI. As such, the experiment is limited by the Fourier coefficients of the binary deactivation patterns, and not by local switching contrast. This diffraction efficiency is obtained at a pump fluence of 4–5 mJ/cm^2^, and could be realized at lower fluence in future experiments if a large area is pumped without the spatial *t*_0_ variation that introduced a deactivation contrast gradient. Based on the spatial extent analysis, we conclude that at least one meta-atom is required to initiate TH deactivation (leading to the minimum resolution of a single meta-atom), with a transition region to complete deactivation that spans two meta-atoms. We assume no role of excited carrier diffusion in the measured spatial resolution, due to the isolation of the poly-Si meta-atoms, and as the relevant diffusion time scales are larger than the duration of the IR probe pulse.^38^

## Conclusion

In summary, we presented all-optical nonlinear beam shaping both in a real-space beam profile and its Fourier-space angular distribution. We achieved this demonstration via pumping a Fano resonant all-dielectric metasurface with a visible pump pulse that broadens and blue-shifts the resonance due to photon absorption-induced excited carriers, thereby deactivating harmonic generation from an IR probe pulse tuned to the Fano resonance. By controlling the time delay between the probe and pump pulse, we revealed the transient resonant response and the impact on the THG. We spatially structured the pump pulse via a DMD to locally deactivate harmonic generation and presented nonlinear real- and Fourier-space shaping, evidencing the coherent nature of TH emission. Furthermore, we found that the spatial pump is effective down to a minimum resolution of a single meta-atom, which is necessary to trigger harmonic generation or deactivation. The deactivation mechanism exhibits a transition region spanning 2 meta-atoms, beyond which complete deactivation is achieved.

Ultrafast modulation speeds are reached on the order of the light pulse itself. An important note is that the onset of the modulation is on the femtosecond time scale, but in optical switching scenarios, the modulation frequency will be limited by the recovery time of the excited free carriers. In our samples, this would limit the modulation frequency to the 200 GHz range (5 ps relaxation times). The present experiments demonstrate the feasibility of beam shaping and pump–probe modulation on a femtosecond time scale as relevant for typical ultrafast laser experiments that range from single-shot up to ca. 100 MHz repetition rate. Engineering of carrier lifetime, i.e., through the introduction of traps, could accelerate recovery times if high-repetition-rate operation is desired.

To better understand the spatial extent of resonance shaping, one could perform hyperspectral mapping of the metasurface, revealing the local optical response as a function of pumping. When carried out at the level of a single meta-atom (or even subunit-cell resolution), this approach could uncover the true nature of resonance modulation at the nanoscale. Furthermore, future research could explore additional avenues for all-optical control of resonances and their influence on harmonic generation or other functionalities of metasurfaces. Recently, it was proposed that structured optical pumping can reveal an asymmetric quasi-BIC from an otherwise symmetric metasurface,^47^ and radiative-loss tuning through temporal symmetry breaking, to optically control resonances, has been experimentally demonstrated.^41^ Together with the present work, these advances highlight the potential of all-optical modulation as a powerful approach for dynamic control of nonlinear light generation and other ultrafast photonic functionalities.

We emphasize that our nonlinear beam shaping approach holds true for any harmonic order. This fact brings UV beam shaping within reach, especially as the mechanisms of nonlinear emission from solids continue to be better understood.^55,57,58^ Given the limited availability of conventional UV optics, the ability to shape the beam profile and control the emission direction at the generation stage could be a game-changer.

## Methods

### Pump–Probe Setup

A 515 nm pump pulse (pulse length 130 fs, ca. 3 nm bandwidth) passes through a delay stage (Newport, power supply: DL-PS, delay line kit: DL-BKIT2U–S-M, stage DL125), in a double-pass setup, to precisely control the time delay between the 515 nm pump and the IR probe. The pump projects a 50× demagnified image (*f* = 200 mm lens, Thorlabs, focuses in the BFP of the 50×, NA 0.8 microscope objective) of the DMD (Texas Instruments, DLP LightCrafter Single DLPC900 EVM, controller: DLPC900, chip: DLP6500) onto our disk-bar metasurface. The individual mirror (pixel) size of the DMD is 7.56 μm, which at 50× demagnification of the setup translates to 151 nm in the sample plane. For a pump wavelength of λ = 515 nm and an NA of 0.8, this produces about 2.1 pixels per diffraction-limited length of λ/2NA ≃ 322 nm. The pump and probe beams are combined right before the objective via a beamsplitter (Thorlabs, BS025). With all pixels on the DMD set to reflect, it effectively functions as a mirror so that the Gaussian beam profile of the pump beam is projected on the metasurface. As all individual pixels are under an angle of ca. 10° across the diagonal in the ON state, this introduces an optical path difference of a few hundred μm (equivalent to ∼0.3 ps) across the pump beam. In all experiments, we maintain an operating repetition rate of 1 MHz. A schematic overview of the experimental setup is presented in the SI.

The TH signal is collected in reflection, where it reflects off a dichroic mirror (550 nm, Edmund Optics) right after the microscope objective, passes through a 1:1 telescope (Thorlabs 2× *f* = 150 mm), a notch filter (Thorlabs NF514–17), and a short pass filter (Thorlabs FESH0500) to remove the pump pulse. Afterward, it is focused on the slit of a grating-based spectrometer (Andor Shamrock 163i, 163 mm focal length, 300 lines/mm, 25 μm slit width, with a CMOS camera Ximea MC124MG-SY-UB) to measure TH spectra, or onto a set of two cameras: one images the real-space plane of the TH emission from the metasurface (Basler acA 1920–40 μm), while the other uses an additional lens (*f* = 200 mm, Thorlabs AC254–200-AB) that focuses in the BFP of the objective to image the Fourier-space plane to study the angular distribution of the TH emission (Thorlabs CS2100M-USB).

## Supplementary Material



## Notes

LINK I. Schematic experimental setup, II. Transient IR probe spectra, III. Diffraction efficiency calculations, IV. Temporal coupled-mode theory and code (PDF)
