# Supplementary material for: All-Optical Nonlinear Real and Fourier-Space Shaping with All-Dielectric Fano Resonant Metasurfaces
Source: ACS Nano. 2026 Feb 6;20(6):4845–54. doi: 10.1021/acsnano.5c16823 (PMC12918718; doi:10.1021/acsnano.5c16823)
Supplement: Supplementary file 1 [file nn5c16823_si_001.pdf]

Supporting Information: All-Optical Nonlinear Real and Fourier Space Shaping with All-Dielectric Fano Resonant Metasurfaces

Falco Bijloo,[†,‡] Masha Ogienko,[†,‡] Arie J. den Boef,[†,¶,§] Peter M. Kraus,[†,¶] and A. Femius Koenderink[*,‡]

†*Advanced Research Center for Nanolithography, Science Park 106, 1098 XG Amsterdam, The Netherlands*

‡*Department of Physics of Information in Matter and Center for Nanophotonics, NWO-I Institute AMOLF, Science Park 104, 1098 XG Amsterdam, The Netherlands*

¶*Department of Physics and Astronomy, and LaserLaB, Vrije Universiteit, 1081 HV Amsterdam, The Netherlands*

§*ASML Netherlands B.V., 5504 DR Veldhoven, The Netherlands*

E-mail: f.koenderink@amolf.nl

pages: 18

figures: 10

I. SCHEMATIC EXPERIMENTAL SETUP

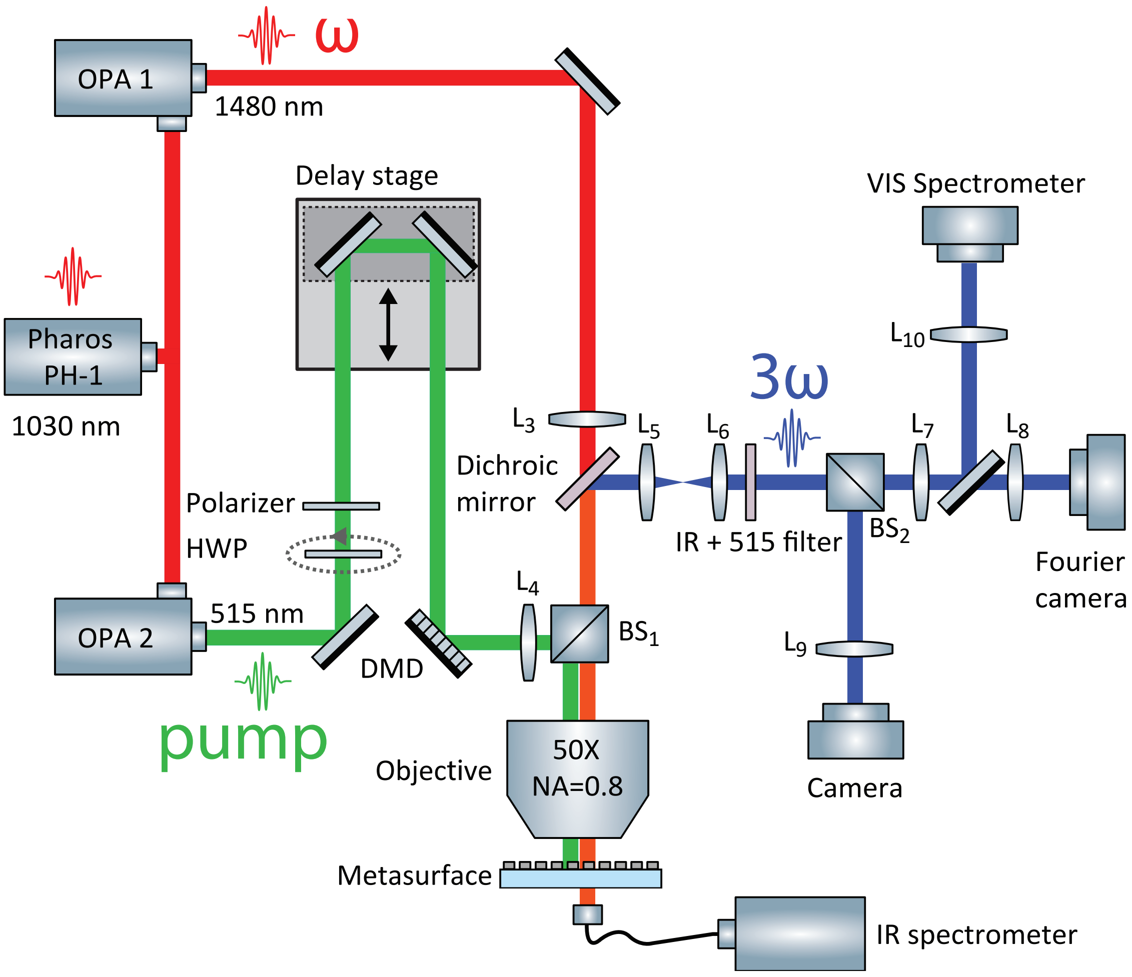

Figure S1.1: Schematic of the experimental setup used for structured TH deactivation experiment. The details of the components are described in the main text.

The components within the experimental setup are described in detail in the main text; here, we provide a schematic illustration for clarity (Fig. S1.1). The colors in the schematic are used symbolically to ease the understanding and reflect the real colors in the experiment. The IR pulse generates TH light in the metasurface, which, due to the reflective setup, retraces the same optical path (indicated in orange) back toward the dichroic mirror. The dichroic mirror then guides the TH signal into the collection path (shown in blue). In practice, the 515 nm pump pulse is also reflected from the metasurface and follows the same return path as the TH signal, but it is blocked by a notch filter, which represented in the

schematic as the '515 filter'.

II. TRANSIENT IR PROBE SPECTRA

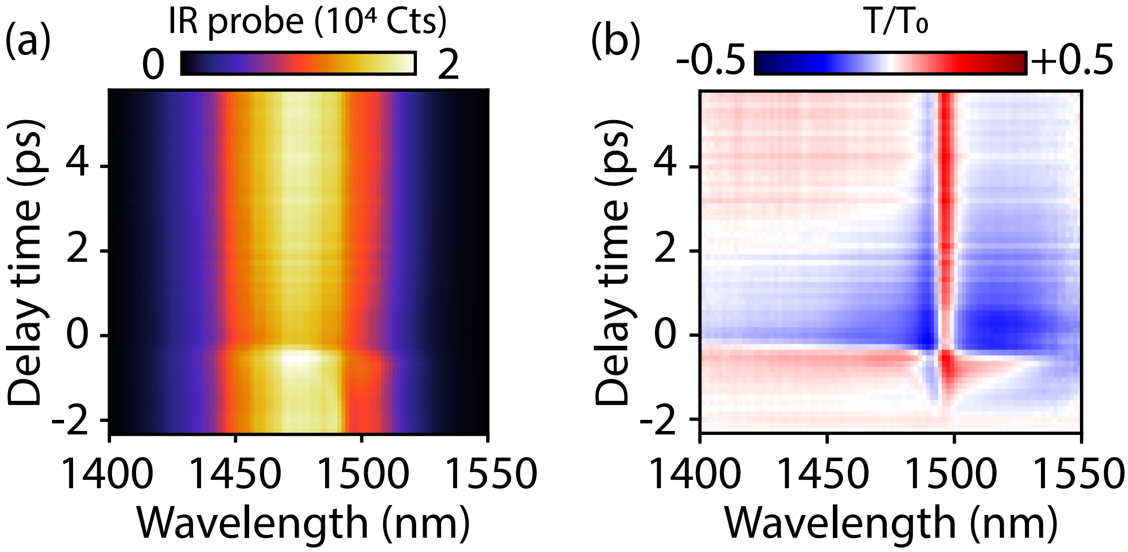

Figure S2.1: a) Transient IR probe spectra (x-axis) for a delay time trace (y-axis). b) Normalized IR transmittance ratio T/T_0, where T_0 is the transmittance spectrum at $t = -2$ ps.

For researchers interested in the complete IR probe transmission data, Fig. S2.1a) displays the IR transmission spectra (x-axis) as a function of pump–probe time delay (y-axis). This probe signal corresponds to the same measurement shown in Fig. 2 in the main text. Additionally, Fig. S2.1b) presents the transient IR transmittance, plotted as the normalized ratio T/T_0, where T_0 is the transmittance spectrum at $t = -2$ ps, to highlight changes.

As an example, Fig. S2.2 presents IR probe reference (*i.e.*, the IR excitation pulse) and IR probe spectrum and corresponding transmittance, defined as the ratio of IR probe to reference, for time delays: $\Delta t = -2$ ps (before temporal overlap) and at $\Delta t = 0$ ps (at temporal overlap).

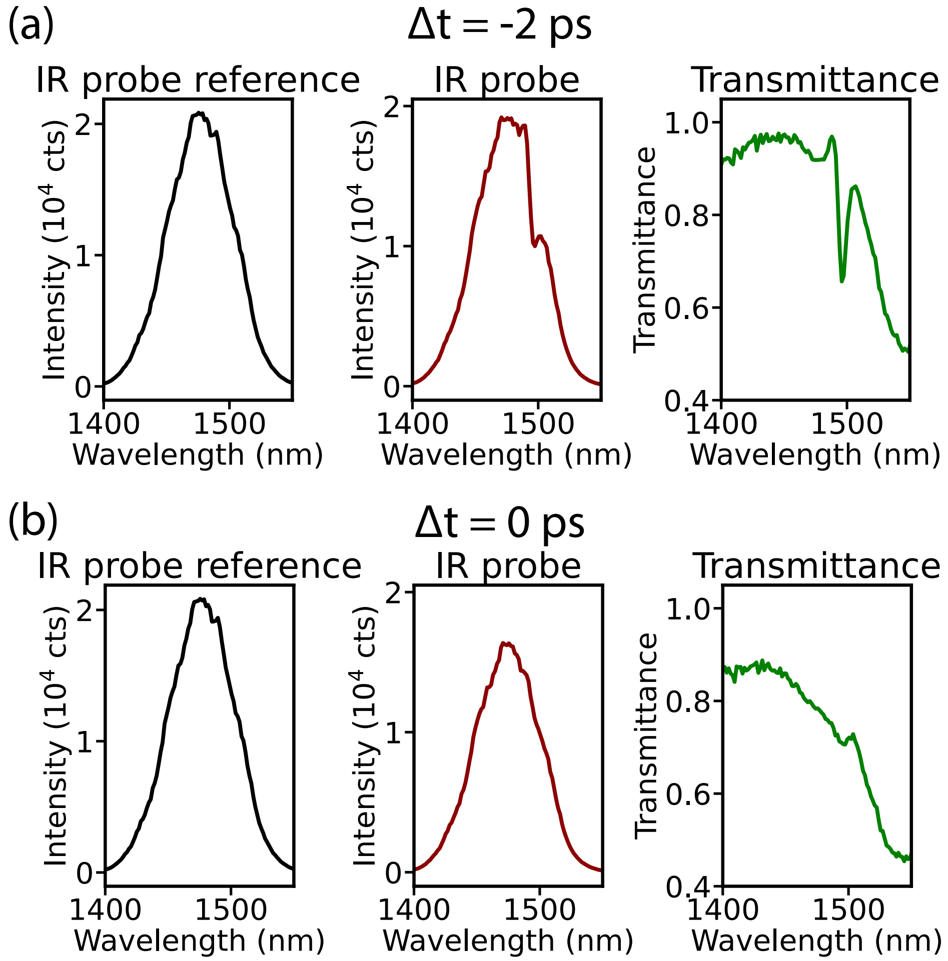

Figure S2.2: Example cross-cuts or the IR excitation pulse (left), IR transmission (middle) and IR transmittance before temporal overlap at delay time $\Delta t = -2$ ps (a) and at overlap $\Delta t = 0$ ps (b).

III. DIFFRACTION EFFICIENCY CALCULATIONS

To assess whether the reduced diffraction efficiency observed below a single meta-atom in the experiment (Fig. 5f in the main text) arises from limitations in deactivation capability or is intrinsic to the nature of convolved 2D grids, we perform a numerical calculation. In this approach, all quantities are redefined in terms of discrete "pixels", meaning that the full analysis presents discrete Fourier transforms (which might lead to artifacts). Since the DMD generates a series of 2D grid patterns with varying gap linewidths, we interpret these gap widths as integer pixel values. All spatial dimensions are subsequently converted to this pixel-based scale. Considering the demagnification of the pump pattern onto the sample (one DMD pixel corresponds to 151 nm in the metasurface plane), the effective number of pixels per metasurface pitch is calculated as 955 [nm] / 151 [nm/pixel] $\sim$ 6 pixels.

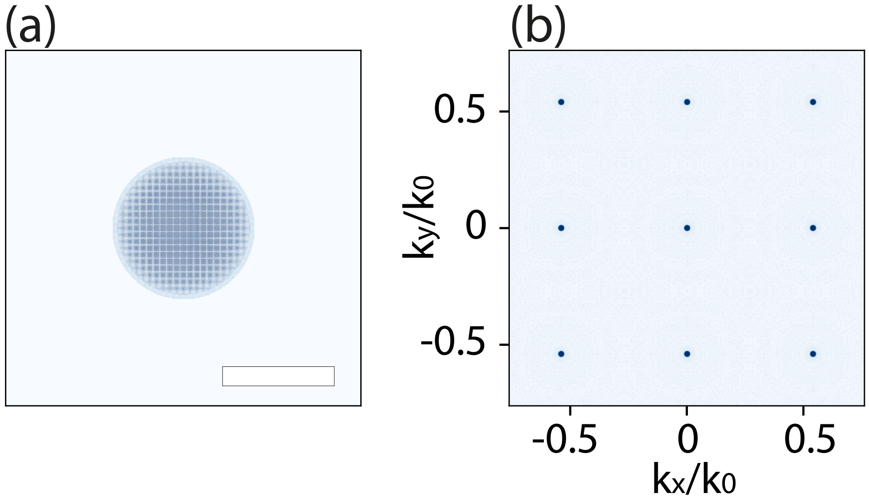

Figure S3.1: Assumed real-space 2D grid with metasurface pitch. b) Discrete Fourier transform yields 'diffraction' orders.

Starting from a 2D grid with a lattice spacing of 6 pixels, we impose a circular Gaussian mask to mimic the spatial Gaussian profile of the excitation beam, shown in Fig. S3.1a).

The discrete Fourier transform (DFT) of the 'metasurface' lattice yields discrete diffraction orders at similar k-values as the experiment (Fig. S3.1b). The second grid, corresponding to the block pattern, is a binary mask with variable gap linewidth w ranging from 0 to 50

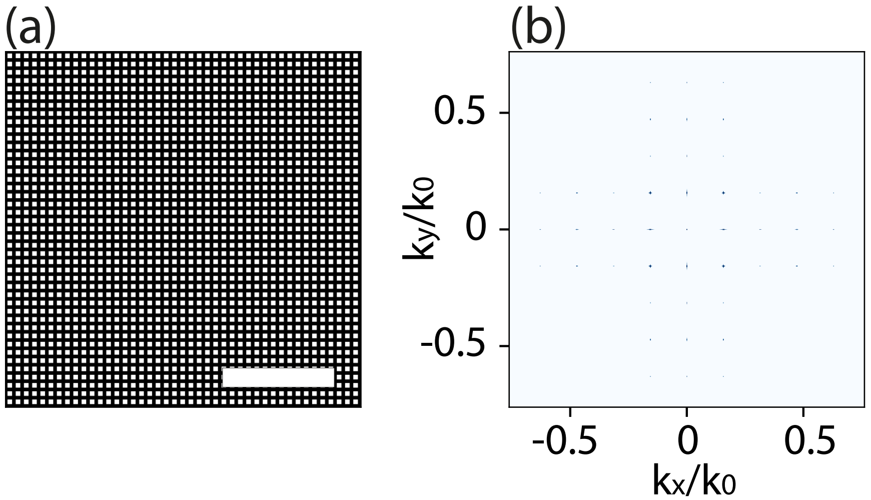

Figure S3.2: Binary pump grid used in the experiment for projection on the DMD, and in the calculation as input for a DFT to obtain the Fourier pattern, which is presented in (b).

pixels (Fig. S3.2a, example at $w = 22$ pixels), that represents the real space DMD pump pattern. Its Fourier transform (Fig. S3.2b) is then multiplied with the Fourier pattern of the metasurface lattice to produce the convolved diffraction pattern (Fig. S3.3a).

Following the same procedure as in the main text, we sum over a small band in k_x and plot k_y versus gap linewidth w (Fig. S3.3b).

Finally, we sum the intensity in each satellite diffraction orders (both of experimental data as numerical calculation) to compare the onset of TH deactivation, presented in Fig. S3.4. The experimental data show that TH signal in the satellite orders emerges only once the deactivation area reaches a single meta-atom, while the calculated intensity ramps up from $w = 0$ μm. This evidences that a minimum deactivation resolution of one meta-atom is experimentally required, in contrast to the calculation, where gradual modulation is possible even below this scale. Furthermore, the TH power flow per single diffraction order is at the level of a few percent (Fig. S3.4a). We note that this is in excellent accord with Fourier optics theory (structure factor form factor analysis) for the same patterns (Fig. S3.4b), and as such our experiment is limited by the Fourier coefficients of the binary patterns that we offer, and not by local switching contrast.

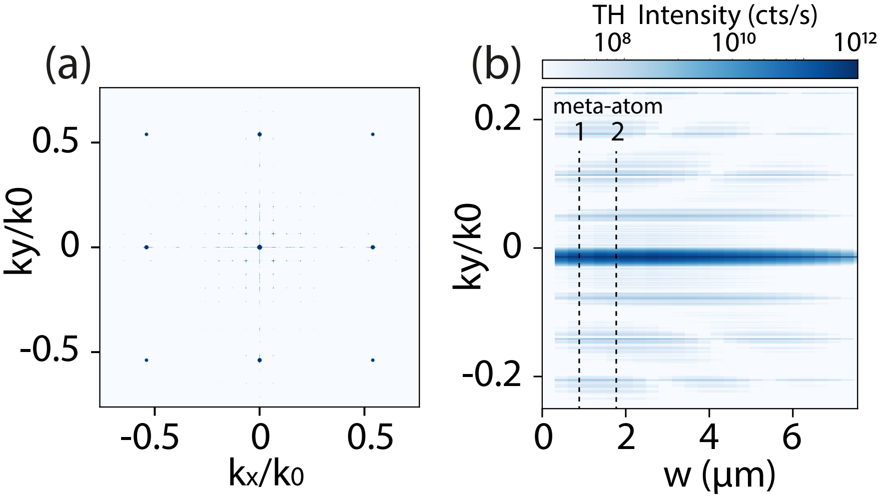

Figure S3.3: Calculated Fourier space of the convolution between the 'metasurface' emission and the binary pump grid, revealing clear discrete diffraction orders as well as satellite features arising from the spatial modulation. b) Calculation of k_y as a function of gap linewidth w, analogous to the experimental data shown in Fig. 5f) in the main text. In the numerical calculation, TH intensity already flows into the diffracted satellite orders below 1 meta-atom.

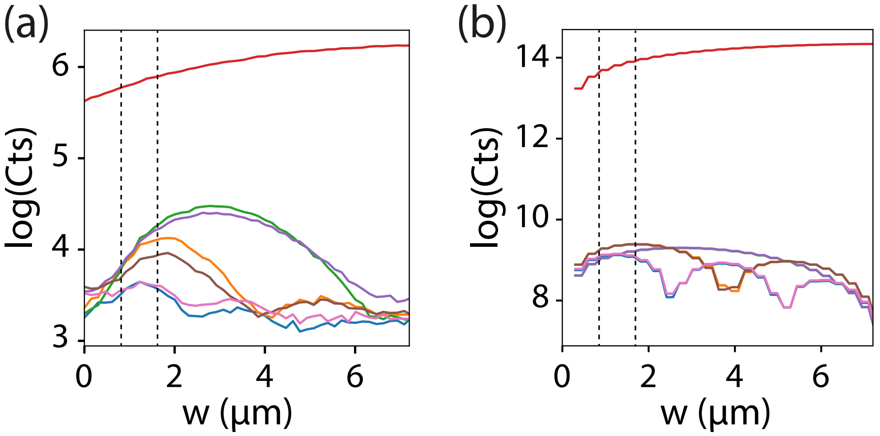

Figure S3.4: Summed intensities in the diffracted satellite orders (red: 0, green/purple: +1/-1, orange/brown: +2/-2, pink/blue: +3/-3) for experiment (a) and calculation (b). Where almost no TH intensity flows in the diffracted satellite orders below 1 meta-atom experimentally, the calculation shows a steady increase well below 1 meta-atom.

IV. TEMPORAL COUPLED-MODE THEORY

To rationalize the transient (ultra-fast) dynamics of the fundamental resonance under pumping and it's influence on the THG spectrum, we evaluate a simple temporal coupled model that replicates the measured observations in the time domain. We take inspiration from Ref.[1] who suggests to solve

$$\dot{a} + [i\omega_0(t)] + \gamma_{nr}(t) + \kappa]a = \sqrt{\kappa}s(t)$$

for a single switched resonance, with $s(t)$ the driving pulse, and with time varying (to be inserted ad hoc into the model) resonance frequency and damping. This equation derives from time dependent coupled-mode theory.

For consistency with our past work,[2] we use a model for Fano resonant systems written in second order instead of first order which reads

$$\begin{aligned} \ddot{a} + (\kappa_{WG} + \kappa_l)\dot{a} + \omega_B^2 a - i\kappa_{12} b &= 2\sqrt{\kappa_{WG}}\dot{s}(t) \\ \ddot{b} + \kappa_F \dot{b} + \omega_F^2 b + i\kappa_{12} a &= 0. \end{aligned}$$

This is a two-oscillator model, to reproduce the Fano lineshape measured in transmission spectra. Here $\omega_{B,F}$ are sthe bright and Fano (dark) resonance frequency, κ_{WG} the coupling rates to the transmission and reflection channel, κ_F the intrinsic Fano damping rate, and κ_{12} the coupling rate between the modes. The rate κ_l can be used to tune the background line shape, effectively imbuing the bright mode with additional loss on top of the coupling of radiation to the input and output port. Here, it should be noted that the factor 2 in the RHS of the bright oscillator equation coupling rate signifies that this is a two port device (both reflection and transmission). The transmitted field follows as $s(t) - \sqrt{\kappa_{WG}}a$. The dark mode is only addressed indirectly, via the near field coupling κ_{12}.

The Python ODE solver `scipy.solve_ivp` that we use requires sets of first order ODEs

to perform numerical integration on, so we convert the two 2nd order equations to four first order equations through the auxiliary variables $A = \dot{a}$ and $B = \dot{b}$ so that

$$\begin{aligned}
\dot{A} + (\kappa_{WG} + \kappa_l)A + \omega_B^2 a - i\kappa_{12} b &= 2\sqrt{\kappa_{WG}}\dot{s}(t) \\
\dot{a} = A & \\
\dot{B} + \kappa_F B + \omega_F^2 b + i\kappa_{12} a &= 0 \\
\dot{b} = B &
\end{aligned}$$

which in matrix notation reads

$$\frac{d}{dt}\begin{pmatrix} A \\ a \\ B \\ b \end{pmatrix} = \begin{pmatrix} -(\kappa_{WG} + \kappa_l) & -\omega_B^2 & 0 & i\kappa_{12} \\ 1 & 0 & 0 & 0 \\ 0 & -i\kappa_{12} & -\kappa_F & -\omega_F^2 \\ 0 & 0 & 1 & 0 \end{pmatrix} \cdot \begin{pmatrix} A \\ a \\ B \\ b \end{pmatrix} + 2\sqrt{\kappa_{WG}}\dot{s}(t)\begin{pmatrix} 1 \\ 0 \\ 0 \\ 0 \end{pmatrix}.$$

This is directly implemented in Python for use with the `scipy` solver `solve_ivp`. We solve this with the initial condition $a = A = b = B = 0$, and set a Gaussian driving pulse as $s(t) = \exp(-(t - t0)^2/2\Delta t^2)\exp(-i\omega_0 t)$. Our model assumes that only the Fano resonance is switched via

$$\begin{pmatrix} \omega_F(t) \\ \kappa_F(t) \end{pmatrix} = \begin{pmatrix} \omega_F \\ \kappa_F \end{pmatrix} + \begin{pmatrix} \Delta\omega_F \\ \Delta\kappa_F \end{pmatrix} H(t - t_s)\exp(-(t - t_s)/t_{\mathrm{Relax}})$$

where $H(x)$ is the Heavyside step function. Here t_s represents the pump probe delay, and t_{Relax} is a measure for the relaxation time of the switch (due to carrier recombination). The magnitude of the switch $\Delta\omega_F$, $\Delta\kappa_F$ could for instance be estimated from full wave calculations, assuming the Drude model for the dielectric constant change that is achieved. We take inspiration from our measured data (Fig. 2d) in the main text) and set $\Delta\omega_F$ and

$\Delta\kappa_F$ accordingly in order that they correspond to a resonant shift of 7 nm ($\lambda_2 = 1495$ nm to $\lambda_2 = 1488$ nm and a broadening of 300% ($Q = 400$ reduced to $Q = 133$). The relaxation time is set at ~ 1.6 ps.

The strategy is to solve the ODEs numerically (`solve_ivp` standard settings). After solving, we Fourier transform $s(t)$, $a(t)$ and $THG(t) = b(t)^3$. The linear transmission follows as $T(\omega) = |(s(\omega) - \sqrt{\kappa_{WG}}a(\omega))/s(\omega))|^2$ and the THG spectrum follows as $|THG(\omega)|^2$. This uses the simplifying assumption, motivated by the higher Q nature of the dark mode, that the THG signal is strictly due to radiation from the dark mode (of amplitude $b(t)$), although one could easily extend the postprocessing to consider the nonlinear mixing of the bright and dark mode.

In absence of any switching, the resulting transmission reproduces the usual frequency domain result that can be analytically derived as

$$T = \left| 1 - \frac{2i\omega\kappa_{WG}\left(\omega_F^2 - \omega^2 + i\omega\kappa_F\right)}{\left(\omega_F^2 - \omega^2 + i\omega\kappa_F\right)\left(\omega_B^2 - \omega^2 + i\omega\left(\kappa_l + \kappa_{\mathrm{WG}}\right)\right) - \kappa_{12}^2} \right|^2$$

(to derive this take the second order time domain equations, and solve at frequency ω) , and parameters for the model can be obtained by fitting the linear spectroscopy data to the model. Figure S4.1 shows a calculated linear transmittance of a Fano resonance at 1495 nm (a) that produces THG (b), and the switched configurations at t_0, showing similar features to that of the observed experimental behavior presented in Fig. 2a-e) of the main text.

Figure S4.2 presents the full modeled time-trace of the linear transmittance and THG spectrum. The agreement between the results of the model and the measured data is striking, revealing similar shift, broadening and recovery of the fundamental, as well as the influence on the transient THG spectrum (including the strong TH deactivation). Pre-t_0 fringes are observed. Similar dynamics are observed by Ref.[1] These fringes are visible in our experimental data in Fig. 2c) in the main text, around the Fano resonance (1490 nm) for 1 ps before t_0. Within our model, the fact that pre-t_0 features occur can be rationalized from the fact that

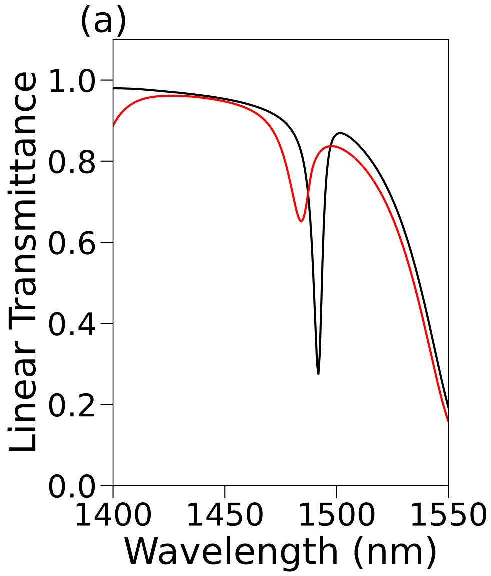

Figure S4.1: Temporal coupled-mode theory model results. (a) Calculated linear transmittance far before t_0 (black curve) reveals a Fano resonance that broadens and blueshifts upon switching at t_0 (red curve). (b) THG spectrum before (at) t_0 in black (red), showing the strong influence of the resonance modulation on THG conversion efficiency.

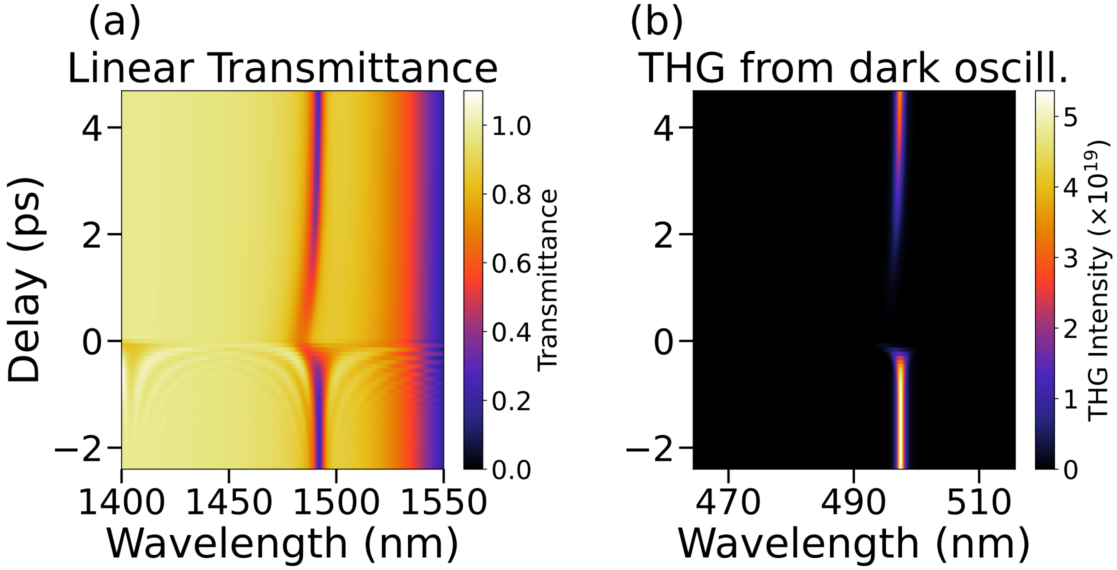

Figure S4.2: Modeled transient results for linear transmittance (a) and the THG (b) for a driving pulse centered at the Fano resonance at 1490 nm. The blue-shift, broadening and recovery of the resonance are clear, as well as the deactivation effect on the THG conversion efficiency. Pre-t_0 dynamics in the fundamental are clearly observed as fringes around the resonance at 1490 nm starting from ~ -1 ps.

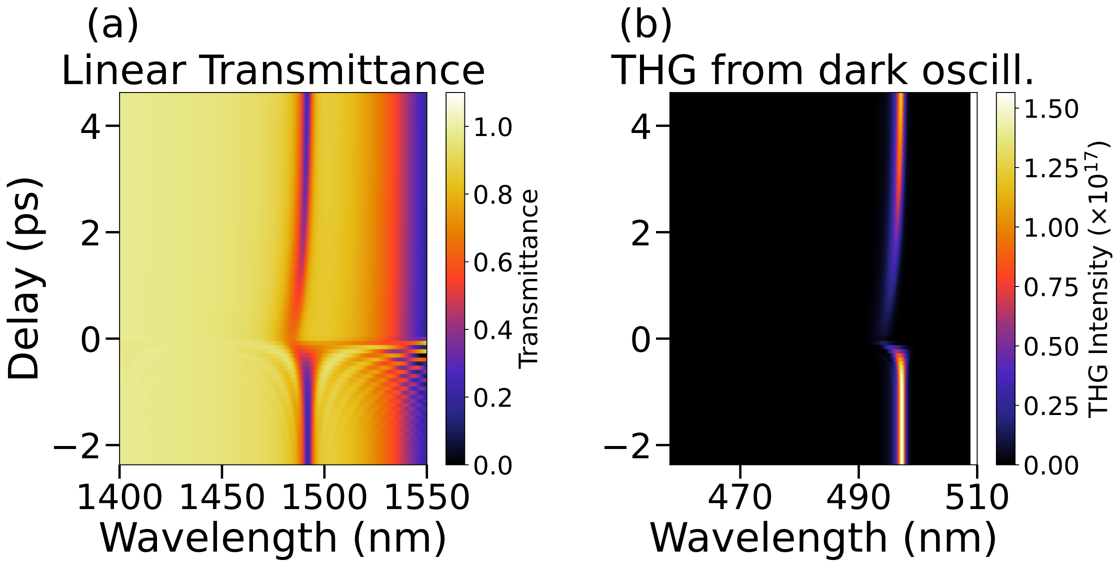

Figure S4.3: Modeled transient results for linear transmittance (a) and the THG (b) for a slightly blue-detuned driving pulse centered at 1465 nm. The transient TH spectrum shows a clear blue-shift that is similarly observed in the experiment (Fig. 2h).

the high Q of the dark mode means that although the probe pulse comes before the abrupt switch, the abrupt switch is within the slow ringdown of the dark mode. As we take $b(t)^3$, we only observe the contribution from the narrow Fano resonator in the THG spectrum. In reality, the situation is more complex, as the broader bright resonator also contributes, resulting in a broader THG spectrum in the experimental data. Moreover, to fully describe the transient dynamics, one must account for interference between these two modes, which further enriches the observed behavior.[2] Furthermore, also the broad resonator should have a switching term for a more realistic scenario. However, these modeled results are clearly capable of describing the important aspects of transient behavior and can lead to further development and understanding of transient resonance shaping and the effect on harmonic generation.

We note that in this simple code the time axis for sampling the solution needs to be long enough to obtain the frequency resolution required (Nyquist theorem) to resolve the Fano resonance, while the sampling needs to be fine enough to resolve both the fundamental and third harmonic.

The experiment for a slightly blue-detuned driving pulse (Fig. 2h) in the main text), revealed a transient TH spectrum that blueshifts into overlap with the pulse and therefore gets slightly enhanced. This observation is clearly reproduced in a calculation for a blue-detuned pulse that is centered at 1465 nm in Fig S4.3.

The full python code that runs for the TCMT model and produces Figs. S4.1-3 is printed below.

```
import numpy as np
from scipy.integrate import solve_ivp
from matplotlib import pyplot as plt

def drivepulse(t,t0,w0,dt):
    ## simple Gaussian pulse. 2nd order CM theory also needs the time derivative
    s=np.exp(-(t-t0)**2/(2*dt**2))*np.exp(-1.0j*w0*t)
    dsdt=s*(-(t-t0)/(dt*dt)-1.0j*w0)

    return s,dsdt

def switchprofile(t,ts,dw,dg,trelax):
    ## assumed switchprofile for the Fano
    profile=(t>=ts)*np.exp(-(t-ts)/trelax)
    dwt= 1.0+dw*profile
    dg=  1.0+dg*profile
    return dwt,dg



def myfft(teval,signal):
    ## used for making frequency plots. Basically a FFT
    ft=np.fft.fftshift(np.fft.fft(signal))
    df=-2.0*np.pi/(teval[-1]-teval[0])

    f=df*(np.arange(0,ft.shape[0])-np.floor(ft.shape[0]/2))
    return f, ft

def converttimetrace(t,drive,sol):
    f,fundam=myfft(t,drive)
    f,oscil1=myfft(t,sol.y[1])
```

```
    f,oscil2=myfft(t,sol.y[3])
    transmission=(np.abs((fundam-np.sqrt(kWG)*oscil1))/np.abs(fundam))
    f,thg=myfft(t,(sol.y[3])**3)

    return f,fundam,oscil1,oscil2,transmission,thg

def TDCMT(wB,kWG,kl,wF,kF,k12,w0,dt,t0,tmin,tmax,tstep, ts,dw,dg,trelax):

    ## time axis for evaluation
    teval=np.arange(tmin,tmax,tstep)

    ## switch profile to renormalize the freq and damping
    a=lambda t: switchprofile(t,ts,dw,dg,trelax)

    ## set up the time dependent cmt
    TDCMTsimple = lambda   t, s: np.dot(np.array([[ -(kWG+kl), -wB*wB,0,-1.0j*k12],
                                        [1.0,0.0,0.0,0.0],
                                        [0, 1.0j*k12,-kF*a(t)[1],-(wF*a(t)[0])**2 ],
                                        [0, 0,1.0,0.0 ]]),s
                            )+2.0*np.sqrt(kWG)*drivepulse(t,t0,w0,dt)[1]*np.array([1.0,0.0,0.0,0.0])

    ## solve the ODE in time
    sol = solve_ivp(TDCMTsimple, [tmin,tmax], [0.0+0.0j, 0,0,0],t_eval=teval)

    ## output the result
    drive=drivepulse(teval,t0,w0,dt)[0]
    f,fundam,oscil1,oscil2,transmission,thg=converttimetrace(teval,drive,sol)


    return f,transmission,np.abs(thg)**2, [[teval,drive,sol.y[1],sol.y[3]],[f,fundam,oscil1,oscil2]]



## parameters for two oscillator model, creating a Fano in transmission
# Run at 1460 for blue-detuned pulse (Fig 2h), run at 1490 for at resonance (Fig 2c)
l0=1490 # in nanometers


wtol = l0/1495 # relative frequency of the pulse to the resonance, the resonance is now at 1495 nm

##Broad brightmode
```

```
wB=wtol*0.961  # normalized frequency (slightly redshifted)
kWG=0.02 # coupling rate to transmission (and reflection) channel
kl=0.01 # additional loss (tunes background lineshape)

## Fano line
wF=wtol*1 # normalized frequency
kF=0.002 # damping rate
k12=0.016 # coupling rate to bright mode. Tune to set visibility

## Driving that generates THG
## Our experiment is at ~1500 nm vacuum wavelength, and 130 fs pulse duration.
## this means omega = 2*pi*c/lambda
## suppose that I work at 1500 nm, so omega =1.256E15. omega0*dt=160
w0=1.0
dt=130*0.44 #(Transform limited pulse)
t0=0

## run time domain solver
tmin=-10*dt  # start of the time axis
tmax=30/kF   # end, should be far enough to resolve ring down
tstep=0.1   # step, should be small enough to resolve third harmonic

## now suppose I switch the pulse
dw=0.007 # relative shift. At 1500 nm this is about 7 nm
dg=3.0  # broadening, relative to the intrinsic linewidth, this is 300%
trelax=2000 # at 1500 nm,  1 ps is about omega trelax = 1256

## to just get the linear unswitched response, use just a single tslist item, which is large and positive
# tslist=np.array([10000.0])
tslist=np.arange(-3000,6000,100)

# Now to do the calculation
T=[]
THG=[]
pulse=[]
for index,ts in enumerate(tslist):
    f,transmission,thg,pulse=TDCMT(wB,kWG,kl,wF,kF,k12,w0,dt,t0,tmin,tmax,tstep, -ts,dw,dg,trelax)
    THG.append(thg)
    T.append(transmission)

T=np.array(T)
```

```
THG=np.array(THG)

## convert axes to optical frequencies
lam=l0/f
# plotrange limited by dynamic range / noise floor of the solver
idx1=np.where((lam>=l0*(1-3.5/dt))*(lam<=l0*(1+3.8/dt)))
idx3=np.where((lam>=l0*0.33*(1-3/dt))*(lam<=l0*0.33*(1+3/dt)))

# and dito for pump probe delay in picoseconds
tslist=tslist/(2.0*np.pi*3E8/(l0*1E-9))*1E12

## extract the data in the significant frequency range
l1=lam[idx1]
T=np.squeeze(T[:,idx1])
l3=lam[idx3]
THG=np.squeeze(THG[:,idx3])

## and for the unswitched and pulse time domain plots
# The last pulse is / should not switched (probe well preceeding the pump)



## Now to make the figures
plt.rcParams.update('font.size': 35,'axes.titlesize': 35,'axes.labelsize': 35,'xtick.labelsize': 30)
plt.rcParams.update('ytick.labelsize': 30,'legend.fontsize': 20)

# Use this for t_0
timestep = 30

tpulse=pulse[0]
t=tpulse[0]
drive=tpulse[1]
oscil1=tpulse[2]
oscil2=tpulse[3]

if index >0:
    T0=T[index,:]
    THG0=THG[index,:]
else:
    T0=T
    THG0=THG
```

```
fig,ax=plt.subplots(1,2,figsize=(16,10))
ax[0].plot(l1,np.abs(T0)**2,color='black',linewidth=2)
ax[0].plot(l1,np.abs(T[timestep])**2,color='red',linewidth=2)
ax[0].set_ylim(0.0, 1.1)
# ax[2].set_xlim(1400,1550)
ax[0].set_xlabel('Wavelength (nm)')
ax[0].set_ylabel('Linear Transmittance')
ax[0].tick_params(axis='both', which='major',length=12,width=1)
ax[0].set_xlim([1400,1550])

ax[1].plot(l3,np.abs(THG0)**2/1e19,color='black',linewidth=2)
ax[1].plot(l3,np.abs(THG[timestep])**2/1e19,color='red',linewidth=2)
ax[1].set_xlabel('Wavelength (nm)')
ax[1].set_ylabel('THG Intensity (10^19)')
ax[1].tick_params(axis='both', which='major',length=12,width=1)
ax[1].set_xticks([470, 490, 510])

fig.text(0.15, 0.9, '(a)', fontsize=34, va='top')
fig.text(0.55, 0.9, '(b)', fontsize=34, va='top')
fig.tight_layout(pad=3.0, w_pad=1.5)
plt.show()

#%%
maxthg=np.max(np.abs(THG)**2)

if index > 0:
    fig, ax = plt.subplots(1, 2, figsize=(16, 9))

    # Linear transmittance
    im0 = ax[0].pcolor(l1, tslist, np.abs(T)**2, vmin=0, vmax=1.1, cmap='CMRmap', rasterized=True)
    ax[0].set_xlabel('Wavelength (nm)')
    ax[0].set_ylabel('Delay (ps)')
    ax[0].set_title('Linear Transmittance')
    ax[0].set_xlim([1400,1550])
    ax[0].set_xticks([1400,1450,1500,1550])
    ax[0].tick_params(axis='both', which='major',length=12,width=2)
    cbar0 = fig.colorbar(im0, ax=ax[0])
    cbar0.set_label('Transmittance',fontsize=22)
    cbar0.ax.tick_params(labelsize=22)

    # THG
```

```
im1 = ax[1].pcolor(
l3, tslist, np.abs(THG)**2 / 1e17,
cmap='CMRmap', vmin=maxthg*0, vmax=maxthg*1/1e17, rasterized=True)
ax[1].set_xlabel('Wavelength (nm)')
ax[1].set_title('THG from dark oscill.')
ax[1].tick_params(axis='both', which='major',length=12,width=2)
# ax[1].set_xlim([1400/3,1550/3])
ax[1].set_xticks([470, 490, 510])

cbar1 = fig.colorbar(im1, ax=ax[1])
cbar1.set_label(r'THG Intensity (10^17)', fontsize=22)
cbar1.ax.tick_params(labelsize=22)
fig.text(0.15, 0.9, '(a)', fontsize=34, va='top')
fig.text(0.55, 0.9, '(b)', fontsize=34, va='top')
fig.tight_layout(pad=3.0, w_pad=1.5)
plt.show()
```

References

(1) Zubyuk, V. V.; Shafirin, P. A.; Shcherbakov, M. R.; Shvets, G.; Fedyanin, A. A. Externally driven nonlinear time-variant metasurfaces. *ACS Photonics* **2022**, *9*, 493–502.

(2) Bijloo, F.; den Boef, A. J.; Kraus, P. M.; Koenderink, A. F. Influence of Driving Pulse Properties on Third-Harmonic Diffraction from Quasi-BIC Metasurfaces. *ACS Photonics* **2025**, *12*, 6620–6630.
